# Benzoic Acid Derivatives as Prodrugs for the Treatment of Tuberculosis

**DOI:** 10.3390/ph15091118

**Published:** 2022-09-07

**Authors:** João P. Pais, Marta Magalhães, Olha Antoniuk, Ivete Barbosa, Raquel Freire, David Pires, Emília Valente, Bernard Testa, Elsa Anes, Luis Constantino

**Affiliations:** 1 Research Institute for Medicines and Pharmaceutical Sciences (iMed.UL), Av. Prof. Gama Pinto, 1649-003 Lisboa, Portugal; 2 Faculty of Pharmacy, University of Lisbon, Av. Prof. Gama Pinto, 1649-003 Lisboa, Portugal; 3 University of Lausanne, 1015 Lausanne, Switzerland

**Keywords:** benzoates, nitrobenzoates, tuberculosis, prodrugs, esterases, mycobacteria

## Abstract

One interesting approach to fight tuberculosis is the use of prodrugs that often have shown improved biological activities over drugs with poor absorption or difficulty to cross membranes. Previous studies demonstrate that weak acids such as benzoic acid, present antimycobacterial activity. Moreover, esters of those acids revealed to be a viable alternative since they may diffuse more easily through the cell membranes. Previously we showed that mycobacteria can easily activate benzoic acid esters by conversion to the corresponding acid. Since Zhang postulated that the activity of the acids can be dependent on their pKa, we set up to synthesize a library of benzoates with different electron withdrawing groups (4-chloro, 2,6-dichloro, 3,5-dichloro, 4-nitro, and 3,5 dinitro), to modulate pKa of the liberated acid and different alkoxy substituents (propyl, hexyl, and phenyl) to modulate their lipophilicity, and tested the activity of the esters and the corresponding free acids against mycobacteria. We also studied the activation of the esters by mycobacterial enzymes and the stability of the compounds in buffer and plasma. We concluded that all the benzoates in our study can be activated by mycobacterial enzymes and that the phenyl and hexyl esters presented higher activity than the corresponding free acids, with the nitrobenzoates, and especially the dinitrobenzoates, showing very interesting antitubercular activity that deserve further exploration. Our results did not show a correlation between the activity and the pKa of the acids.

## 1. Introduction

Tuberculosis (TB) is a disease that has been documented throughout the ages and became rampant with the industrial revolution. Multiple societal factors, such as malnutrition, overcrowding, and lack of hygiene, provided optimal conditions for TB to spread [1,2]. Its causative agent, *Mycobacterium tuberculosis* (MTB) was brought under control by the discovery of antibiotics and modern medicine upon the development of a suitable combination therapy regimens; however, until the COVID-19 pandemic, tuberculosis was the leading cause of death from a single infectious agent, ranking above HIV/AIDS. Moreover, the number of cases increased significantly during the COVID-19 pandemic [3].

Aromatic acid moieties can be identified in food preservatives [4] but also in important antitubercular agents, such as para-aminosalicylic acid (PAS) or pyrazinoic acid, which is the active form of pyrazinamide [5]. Hence, small molecules of aromatic nature and with acid function can be of interest for further study. In fact, the simplest molecule with the desired structural features, benzoic acid (BA), is largely used as a preservative in food industry, and it indeed has demonstrated antimicrobial properties against many microbial agents [6], including mycobacteria [5].

One potential approach to TB therapeutics that remains underexplored is a unique aspect of MTB: it is more susceptible to the generality of weak acids compared with other bacterial species, even closely related mycobacterial species, such as *Mycobacterium smegmatis* [5,7]. Furthermore, it was shown to be impossible to isolate resistant MTB mutants resistant to a range of weak acids such as salicylic acid, benzoic acid, and 4-nonyloxybenzoic acid, and its sensitivity to acids can be enhanced by several stress factors [5]. 

With the emergence of MDR and XDR TB strains, the need for new therapeutics is on the rise, and old antitubercular compounds have re-emerged as possible scaffolds for new therapeutic options [8]. It is with this in mind that our research group has studied benzoic acid and its derivatives for their potential, specifically against *Mycobacterium tuberculosis*. Obtaining prodrug versions of an antibiotic is one attractive method for improving activity or shifting toxicity [9,10]. A prodrug is an inactive version of a drug that is administered to a patient and is activated by an enzymatic or chemical process to expose the active drug [11,12].

The starting point of our study was the testing of Zhang’s hypothesis that the antimycobacterial activity of weak acids can be related to its pKa [5]. Zhang reported that the antituberculous activity of a series of the weak acids appeared to inversely correlate with its pKa. However, the structural diversity of the acids in the original study was large and the range of pKa values was small. We decided to decrease the diversity of the structures studying only derivatives of benzoic acid and using different electrons withdrawing groups to lower the pKa of the compounds over a wider range.

In the study, we also included three esters of each acid, as we found previously that benzoic acid esters showed more activity than the free acid, presumably due to acting as prodrugs [13]. The rational for introduction of the esters as potential prodrugs was that the esters can facilitate entry to MTB bacilli due to its higher lipophilicity, and then be hydrolysed by mycobacterial esterases to the respective acids [10,14]. MTB is an especially attractive target to a prodrug approach since it possesses a rich lipid metabolism with diverse esterase and lipase enzymes [15,16,17]. Furthermore, studies indicate that the cleavage of short-chain and medium-chain esters occurs by different enzymes [18] and, previously, we showed evidence that mycobacteria were able to easily hydrolyse a variety of organic acid esters [19]. Additionally, have found that the activity of pyrazinoic acid was improved by esterification and both the acid moiety and the alcohol moiety of the compounds contribute to their antimycobacterial activity [20]. 

This reenforces the conviction that esters of substituted benzoates containing different electron withdrawing groups in the benzoate moiety to modulate the pKa of the acid can be a viable alternative to improve the activity of the benzoates.

## 2. Results

Table 1 presents the structures of the compounds tested in the present study. We tested a variety of benzoates substituted with electron withdrawing groups X to lower the pKa of the free acid liberated by ester hydrolysis. For each series of derivatives, we tested the free acid R = H, and three potential ester prodrugs (R = hexyl, phenyl, and propyl). 

The activity of the compounds was tested against *M. tuberculosis*, *M. bovis* BCG, and *M. smegmatis*, the activation of the esters by mycobacterial enzymes was also tested.

Since the proposed prodrugs must be able to overcome degradation by mammalian enzymes, we also studied the stability of the compounds in human plasma (and as well as in buffer for rate comparison purposes).

### 2.1. Activity against M. tuberculosis

**Figure 1:**
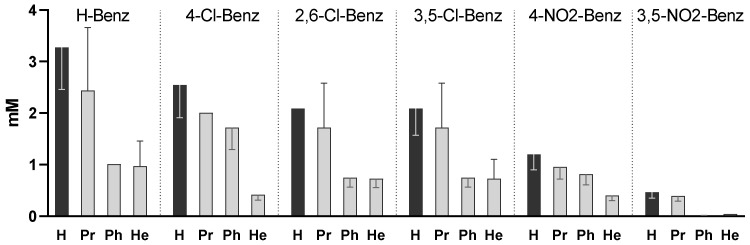
MIC of the free acids and the esters against *M. tuberculosis* (6 series of compounds). Each series has a different electron withdrawing substituent in the benzoyl group (-H, -4-Cl, -2,6-Cl, -3,5-Cl, -4-NO_2_, and -3,5-NO_2_) and is composed of a free acid (H) and three esters: propyl (Pr), phenyl (Ph), and hexyl (He). Data represent the median of 3 independent experiments and the error bars depict the interquartile range.

Figure 1 represents the MIC of all 24 compounds against MTB. The results show that both the aromatic substituent and the alcoxy group influenced the activity.

The simple benzoates (unsubstituted in the benzoyl ring) were the least active compounds, while the most active compounds vs. the unsubstituted benzoates were the 4-dinitrobenzoates (*p* < 0.0005) and 3,5-dinitrobenzoates (*p* < 0.0001).

For all series of compounds (same benzoyl group), phenyl and hexyl esters presented higher activity than the corresponding free acids (*p* < 0.005 for Ph; *p* < 0.0005 for He) indicating that the activity of the free acids was enhanced by esterification, probably by facilitating entrance into the mycobacterial cells. The propyl esterification led to lower MIC values than the corresponding free acid and did not yield statistically significant results. The compounds that presented a lower MIC were the phenyl and hexyl esters of the 3,5-dinitrobenzoate series (0.010 and 0.040 mM, respectively).

**Figure 2:**
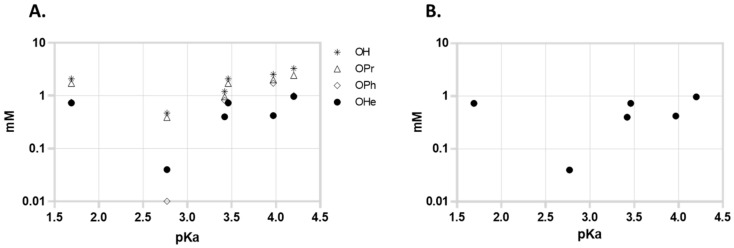
Activity (MIC) against *M. tuberculosis* in function of the pKa of the free acid of the series. (**A**) All compounds, (**B**) most active series (hexyl). Free acids: pKa: 2.6-dichlorobenzoic acid - 1.69; 3,5-dinitrobenzoic acid - 2.77; 3,5-dichlorobenzoic acid - 3.46; 4-nitrobenzoic acid - 3.42; 4-chlorobenzoic acid - 3.97; benzoic acid - 4.20. MIC are the median of 3 independent experiments.

Since Zhang proposed that the activity of the compounds might be related to the pKa of the acids, we represented the MIC of the compounds in function of the pKa of the free acid; however, we could not find a correlation in the aggregate series of the compounds (or in the free acid, hexyl, phenyl, or propyl series considered individually). Figure 2A represents the activity of all compounds and 2B represents the activity of the most active series (hexyl) against MTB in function of the pKa of liberated free acid. The series with a higher pKa of the free acid are the 2,6-dichlorobenzoates (pKa = 1.69) but 3,5-dinitrobenzoates (pKa = 2.77) presented higher activity. The 4-Nitrobenzoate series (pKa = 3.46) also presented slightly higher activity than the 3,5-dichlorobenzoate series (both free acids have comparable pKa values), so as a first observation it seems that the effect of the presence of nitro groups is more important for the activity than the pKa of the liberated acid.

### 2.2. Activity against M. bovis BCG and M. smegmatis

**Figure 3:**
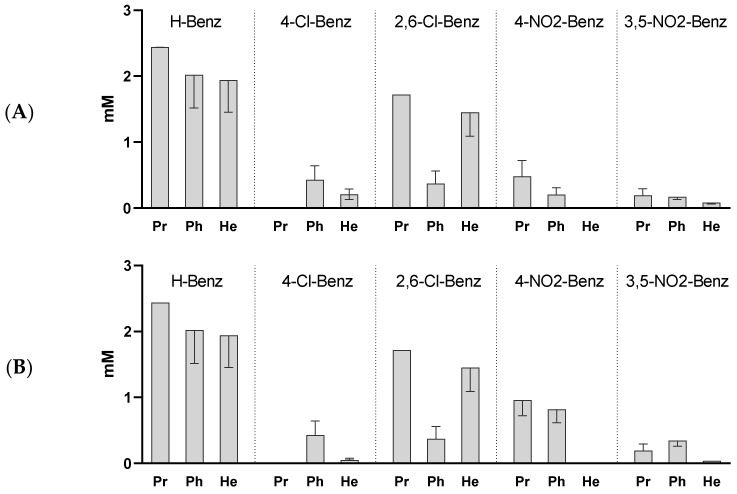
MIC esters under study against *M. bovis* BCG (**A**) and *M. smegmatis* (**B**). Propyl 4-chlorobenzoate was not tested. Data represent the median of 3 independent experiments and the error bars depict the interquartile range.

The activity of the esters against two additional species of mycobacteria, *M. bovis* BCG and *M. smegmatis* are represented in Figure 3. In both cases the observed pattern of activity was similar to the one observed with *M. tuberculosis*. The introduction of electron withdrawing substituents in the aromatic ring of the compounds improved the activity in relation to the unsubstituted benzoates, but no direct correlation could be found between the observed activity and pKa of the acids. Again, we concluded that the more active compounds were the 3,5-dinitrobenzoates (*p* < 0.0001 vs. H-Benz), pointing to the positive effect of this substitution on the activity.

### 2.3. Stability of the Esters in Buffer

**Figure 4:**
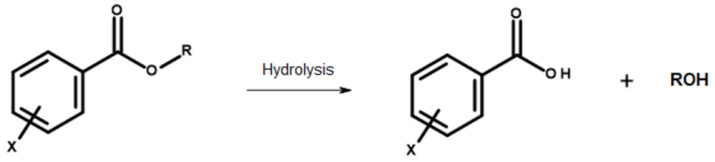
General reaction of the hydrolysis reaction for the benzoic acid derivatives under study.

Hydrolysis of the esters: The main structures discussed in the present work are esters, which are susceptible to hydrolysis by a variety of media (Figure 4). We studied the hydrolysis of the compounds in plasma to check if they can survive plasma transportation and the hydrolysis in mycobacterial homogenate to check if the esters can be activated by mycobacterial enzymes.

In order to evaluate if the chemical hydrolysis performs a significant role in plasma hydrolysis or mycobacterial homogenate activation, we measured the pseudo-first order rate constants at phosphate buffer pH 7.4 with 2% and 20% acetonitrile. The compounds show k_obs_ values between 2.6 × 10^−3^ h^−1^ and 3.7 × 10^−1^ h^−1^, a value significantly below the values of k_obs_ obtained in the biological media studies (see below) meaning that chemical hydrolysis does not contribute significantly to the rates observed in the biological media. The chemical hydrolysis was also evaluated at pH 5.99, k_obs_ values ranged between 5.6 × 10^−3^ h^−1^ and 2.3 × 10^−1^ h^−1^, meaning that the prodrugs present similar stability in those conditions.

### 2.4. Stability of the Esters in Plasma

The esters in the present study were designed to be evaluated as prodrugs of the respective free acid, so it is important to evaluate if the ester can survive plasma hydrolysis and have a chance of delivering the fee acid only inside the mycobacterial cell after cell entry. For plasma stability evaluation we incubated each ester with 80% plasma and measured the pseudo first order rates for the hydrolysis of the compound yielding the respective free acid.

The results are represented in Table 2. The rate of hydrolysis in plasma is affected by the introduction of electron withdrawing groups in the aromatic ring. All substituted compounds demonstrate k_obs_ values lower that the corresponding H-benzoate. Souza et al. [21] found that the introduction of bromine in the aromatic ring caused a small increase in the hydrolysis rate of ethylbenzoate in rat plasma, an opposite effect to what is here observed. This effect observed in our study is, probably, mainly due to a decreased affinity of human plasma esterases vs. rat plasma esterases for these substrates. Nevertheless, the increased stability that we observed in plasma can be of utility in the design of prodrugs.

### 2.5. Activation of the Esters in Mycobacterial Homogenate

**Figure 5:**
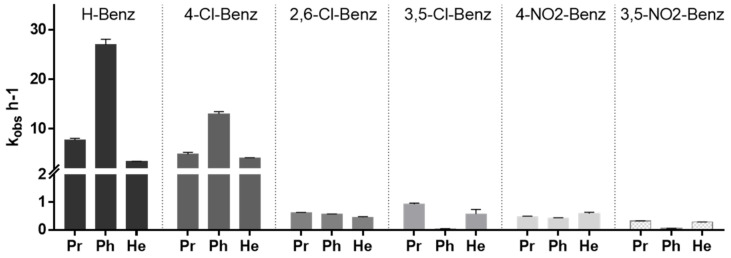
Pseudo first order rate constants for the activation by hydrolysis of the esters under study in *M. smegmatis* homogenate.

Since the compounds were designed to liberate free acid inside the mycobacterial cell, it is important to evaluate if the compounds can be in fact activated by mycobacterial enzymes. For that we prepared a *M. smegmatis* homogenate, incubated the esters in the media, and calculated the pseudo first order rate of the hydrolysis by quantitating the liberation of the free acid. Results are represented in Figure 5 and it can be observed that the more active compounds are the ones that are activated more slowly to the respective free acid.

## 3. Discussion

The results here revealed that the weak acids tested possess antimycobacterial activities against *M. tuberculosis*, *M. bovis* BCG, and *M. smegmatis*. The introduction of electron withdrawing groups in the aromatic ring led in all cases to an increase in activity although the activity is not directly correlated with the pKa of the acid. 

Another relevant result was that for all series, the effect of substitution in the carboxylic acid group leads also to an improvement in activity against MTB. Weak acids may not be easily absorbed through the gastrointestinal tract, bind to serum proteins, or have difficulties in permeating though mycobacterial cells since they are ionized at physiological pH [5]. To circumvent this potential problem, it may be useful to make precursors of weak acids such as ester prodrugs [22,23,24]. To show activity the weak acid precursors must be hydrolysed by enzymes present in MTB, which is known to contain a range of esterases [10,16].

For each of the series studied, the general order of activity is hexyl ester > phenyl ester > propyl ester> free acid showing that at least for some structures, esters can be a good approach to deliver free acids with antitubercular activity.

On a further note, the esters of 3,5-dinitrobenzoic acid revealed to be the most active and promising compounds against MTB, exhibiting MIC values of 20 µM–40 µM for the dinitro phenyl and hexyl benzoates, respectively, far lower than all the other compounds tested and within concentrations lower than those found using pyrazinamide in the same conditions [20,25].These results can be the consequence of a specific role played by the nitro group, such as being the only ionic group tested, resulting in a putative diverse mode of action for these compounds.

When we tested the esters against *M. bovis* BCG and *M. smegmatis* the compounds also revealed antimicrobial activity being once again the dinitro derivatives the ones with the most interesting activity.

The view that the nitro derivatives may have a mode of action distinct from the other weak acids may also be supported by the fact that these esters are the more active but also the less prone to hydrolysis to the free acid in mycobacterial homogenate. As the activity is not directly related to the behaviour of the ester as a prodrug it may indicate that the compound is indeed acting as a drug with a distinct mode of action. Additionally, given the difference in antitubercular activity observed, it is even possible that the 3,5-dinitrobenzoic acid and its derivatives are acting by a different mechanism from the other weak acids.

The standard incubation time for MIC value determination in *Mycobacterium tuberculosis* is at least 10 days. If we use mycobacterial homogenate kobs values (Table 2), as a proxy for the stability of the compounds in the conditions of MIC determination, we can notice that kobs values vary between 27 ± 1.1 h^−1^ and 0.04 ± 0.003 h^−1^. So, half-life for the less stable compound is 0.026 h and for the more stable compound half-life is 17.3 h. It is possible that by day 10, all esters under study can be fully hydrolysed and that the activity can be due to liberation of the acid.

One interesting discussion regarding the more active compounds and the possibility that they may act by a different mechanism is whether 3,5-dinitrobenzoic acid is the active molecule or if 3,5-dinitrobenzoate esters are active per se. The dinitro benzoates presented half-lives between 2.1 h and 13.1 h in mycobacterial homogenate so it is also possible that the activity is due to the free acid liberated by hydrolysis. However, if we compare the activity of 3,5-dinitrobenzoic acid with the 3,5-dinitrobenzoate esters (Figure 1), we can notice that the hexyl and phenyl present significant higher activity than the free acids. We conclude that either (i) the R groups have a positive effect on the interaction of the drug with its target, or (ii) the free acid is the active drug, but the esters are acting as prodrugs, facilitating its absorption. In either case the esterification of the 3,5-dinitrobenzoic acid with He or Ph groups had a positive effect in the activity. With this in mind, and in order to gain insight into their mode of action, it is necessary to study the role of the alcohol moiety for the bioactivity and optimize it, and to understand if the increased antitubercular activity is due to a synergistic action of the products of hydrolysis or due to the entire molecular entity. We plan to improve the alcoxy group, and procure derivatives with different susceptibility to hydrolysis. The fact that 3,5-dinitrobenzoates are very stable in plasma also makes the compounds very interesting because they may be suitable for in vivo administration and able to survive the transport phase.

## 4. Materials and Methods

*Materials:* Balanced salt solution, phosphate-buffered saline (PBS), Dulbecco’s modified Eagle’s medium (DMEM), and L-glutamine were purchased from Invitrogen. Sodium dodecyl sulfate (SDS), Triton X-100, benzoic acid, 4-chlorobenzoic acid, 4-chlorobenzoyl chloride, 3,5-dichlorobenzoic acid, 3,5-dichlorobenzoyl chloride, 2,6-dichlorobenzoic acid, 2,6-dichlorobenzoyl chloride, 4-nitrobenzoic acid, 4-nitrobenzoyl chloride 3,5-dinitrobenzoic acid, 3,5-dinitrobenzoyl chloride propylbenzoate, phenylbenzoate, and hexylbenzoate were purchased from Sigma-Aldrich Quimica SA., the remaining esters were obtained from in-house library or were prepared by the general procedure described below. Middlebrook 7H10 agar was purchased from Difco. Microwell tissue culture plates were purchased from Nunc. They then were prepared in stock solutions of 8 mg/mL in dimethyl sulfoxide (DMSO), and further diluted in Middlebrook 7H9 medium containing oleic acid, albumin, dextrose, and catalase (OADC; Sigma-Aldrich).

*Ester synthesis:* A solution of the appropriate acyl chloride (1.2 mmole per mmole of alcohol) in dichloromethane was added dropwise to a solution of corresponding alcohol and triethylamine (1 mmole per mmole of acid chloride) in dichloromethane at 0 °C. When the reaction was complete (as assessed by TLC using hexane:ethyl acetate, 5:1 to 1:1,or ethyl acetate as eluent) the reaction mixture was filtered and the filtrate washed successively with 10 mL of distilled water and with 15 mL of saturated sodium bicarbonate solution. The dichloromethane solution was subsequently dried, and the solvent evaporated. The residue was purified by column chromatography (silica gel 60) using hexane: ethyl acetate, 5:1 to 1:1, or ethyl acetate as eluent. The characterization of all synthesized compounds is available in the Supplementary Materials.

*Bacterial strains: M. tuberculosis* H37Rv (ATCC 27294), and *M. bovis* BCG (CIP 105050) were used for MIC evaluation. *M. smegmatis* (mc2 155) was used for homogenate preparation and for MIC determination.

*Plasma stability:* Pooled human plasma (960 µL), pH 7.4 phosphate buffered saline (216 µL), 18 µL ACN and 6 µL of a 10^−1^ M stock solution of the prodrug in ACN were mixed in a 2mL vial. The suspensions were incubated at 37 °C and 50 µL aliquots were removed, mixed with 450 µL of ACN: ZnSO4 1% 1:1 and centrifuged. The supernatant was removed and analysed by HPLC, the remaining substrate and the correspondent acid measured.

*Buffer stability:* 1176 µL pH 7.4 phosphate buffer or pH 5.9 phosphate buffer, 18 µL ACN and 6 µL of a 10-1 M stock solution of the prodrug in ACN were mixed in a 2mL vial. The solution was incubated at 37 °C and 50 µL aliquots were removed, mixed with 450 µL of ACN: H2O 1:1. The solution was analysed by HPLC, the remaining substrate and the correspondent acid were both measured.

*HPLC analysis:* Two HPLC systems were used. The buffer and plasma stability studies were performed in an HPLC system with a photodiode detector L-3000 Photo Diode Array Detector, Merck-Hitachi L-6000 pump, Merck-Hitachi D-2500 integrator, and Merck RP-8 column. Activation studies were performed in an HPLC system with a UV detector Merck-Hitachi UV-L7400, Merck Hitachi L-7100 pump, an auto sampler Merck-Hitachi AS 2000, and a Merck-Hitachi D-2500 integrator and Merck RP-8 column. The eluant was a mixture of acetonitrile (60%/70%) and aqueous phosphate buffer with 5% of KH2PO4/H3PO4 0.025M (40%/30%). The flow rate was always 1 mL/min and wavelength was set at 230 nm. All quantifications were evaluated using calibration curves.

*MIC determination:* The MIC of each compound against mycobacteria was evaluated using a methodology based on the reference method endorsed by the European Committee on Antimicrobial Susceptibility Testing (EUCAST) [26] using the broth dilution method in 96-well plates serial dilutions by testing drug concentration ranging from 800 to 1.56 µg mL^−1^. These essays were performed in Middlebrook 7H9 medium for *M. smegmatis* or supplemented with OADC when using MTB or *M. bovis*. The load of mycobacteria used corresponding to approximately 10^5^ CFU of organisms/mL Incubation was performed for 10 days at 37 °C. The results are the median calculated from three independent experiments and corresponds to concentration where no visible turbidity was observed. Data dispersion is represented by the interquartile range. Statistical analysis was performed in GraphPad Prism 9. Statistical significance was tested by two-way ANOVA test and multiple comparisons were performed by Tukey’s post-hoc test. Kanamycin, DMSO, and a well with no drug were used as controls.

*Mycobacterial homogenate:* A crude whole mycobacterial homogenate was prepared according to [19]. A culture of exponentially growing *M. smegmatis* ATCC607 variant mc2 155 with an O.D.600 nm of 0.8–1.0 was harvested by centrifugation at T = 4 °C for 10 min, washed and re-suspended in pH = 7.4 phosphate buffer saline PBS (25 mL for each 750 mL of the initial growing broth). The bacterial homogenate was prepared using an ultra-sound probe with a sequence of five cycles of 2 min each. The homogenate was afterwards divided in 1 mL portions and kept at −80 °C until use. Total protein concentration was 1.4 mg mL^−1^.

*Conditions of incubations and preparation of samples:* The initial substrate concentration was 5 × 10^−^^4^ M in all stability assays. All incubations were carried out at pH 7.4 and 37 °C under agitation using the phosphate buffer described above as diluting agent. The levels of dilution of the mycobacterial homogenate (20%) and human plasma (80%) in the incubates were chosen following preliminary assays to ensure pseudo-first order kinetics in the hydrolysis of benzoates. Acetonitrile (2%) was used in all studies to ensure adequate solubilization of the substrates. The benzoates were added from 10 to 1M acetonitrile stock solutions. After incubation, aliquots of 50 μL were taken into vials containing 450 μL of a 1:1 solution of 1% zinc sulfate and acetonitrile, mixed in a vortex and centrifuged for 10 min at 15,000 rpm. The supernatant was then injected into the HPLC and analysed for quantification of benzoic acid and remaining benzoate. All quantifications were performed using calibration curves.

## Figures and Tables

**Table 1:** Structure of the compounds under study, MW, pKa of the free acid, and MIC against *M. tuberculosis*. The ionization constants (pKa) were predicted using ACD/pKa (ACD/Labs, version 12; Advanced Chemistry Development: Toronto, Canada) and are in accordance with that described in their pKa data base (ACD/pKa DB)”.

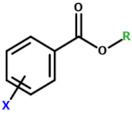					
Compound	X	R	MW	pKa	MIC (mM)
H-Benz-OH	H	H	122.1	4.20	3.28
H-Benz-OPr	H	C_3_H_7_	164.2		2.44
H-Benz-OPh	H	C_6_H_5_	198.2		1.01
H-Benz-OHe	H	C_6_H_13_	206.3		0.97
4-Cl-Benz-OH	4-Cl	H	156.6	3.97	2.55
4-Cl-Benz-OPr	4-Cl	C_3_H_7_	198.6		2.01
4-Cl-Benz-OPh	4-Cl	C_6_H_5_	232.6		1.72
4-Cl-Benz-OHe	4-Cl	C_6_H_13_	240.6		0.42
2,6-Cl-Benz-OH	2,6-Cl	H	191.0	1.69	2.09
2,6-Cl-Benz-Pr	2,6-Cl	C_3_H_7_	233.1		1.72
2,6-Cl-Benz-OPh	2,6-Cl	C_6_H_5_	267.1		0.75
2,6-Cl-Benz-OHe	2,6-Cl	C_6_H_13_	275.1		0.73
3,5-Cl-Benz-OH	3,5-Cl	H	191.0	3.46	2.09
3,5-Cl-Benz-OPr	3,5-Cl	C_3_H_7_	233.1		1.72
3,5-Cl-Benz-OPh	3,5-Cl	C_6_H_5_	267.1		0.75
3,5-Cl-Benz-OHe	3,5-Cl	C_6_H_13_	275.1		0.73
4-NO_2_-Benz-OH	4-NO_2_	H	167.1	3.42	1.20
4-NO_2_-Benz-OPr	4-NO_2_	C_3_H_7_	209.2		0.96
4-NO_2_-Benz-OPh	4-NO_2_	C_6_H_5_	243.2		0.82
4-NO_2_-Benz-OHe	4-NO_2_	C_6_H_13_	251.2		0.40
3,5-NO_2_-Benz-OH	3,5-NO_2_	H	212.1	2.77	0.47
3,5-NO_2_-Benz-OPr	3,5-NO_2_	C_3_H_7_	254.2		0.39
3,5-NO_2_-Benz-OPh	3,5-NO_2_	C_6_H_5_	288.2		0.01
3,5-NO_2_-Benz-OHe	3,5-NO_2_	C_6_H_13_	296.2		0.04

**Table 2:** Pseudo first order rate constants for the hydrolysis of the benzoates in 80% human plasma and in mycobacterial homogenate. Data are representative of three independent experiments and values are expressed in mean ±SD.

	k_obs_ (h^−1^)	
Compound	Plasma	M. homogenate
H-Benz-OPr	2.03 ± 0.58	7.8 ± 0,3
H-Benz-OPh	78.6 ± 0.68	27,0 ± 1,1
H-Benz-OHe	1.10 ± 0.05	3.4 ± 0.1
4-Cl-Benz-OPr	0.64 ± 0.01	4.9 ± 0.4
4-Cl-Benz-OPh	1.26 ± 0.05	13.0 ± 0.5
4-Cl-Benz-OHe	0.24 ± 0.005	4.1 ± 0.1
2,6-Cl-Benz-Pr	0.017 ± 0.001	0.63 ± 0.004
2,6-Cl-Benz-OPh	0.007 ± 0.0001	0.57 ± 0.006
2,6-Cl-Benz-OHe	0.024 ± 0.002	0.46 ± 0.02
3,5-Cl-Benz-OPr	0.16 ± 0.02	0.93 ± 0.004
3,5-Cl-Benz-OPh	0.57 ± 0.03	0.040 ± 0.003
3,5-Cl-Benz-OHe	0.026 ± 0.004	0.58 ± 0.16
4-NO_2_-Benz-OPr	1.32 ± 0.16	0.48 ± 0.02
4-NO_2_-Benz-OPh	0.065 ± 0.003	0.43 ± 0.01
4-NO_2_-Benz-OHe	0.025 ± 0.0003	0.59 ± 0.05
3,5-NO_2_-Benz-OPr	0.057 ± 0.002	0.32 ± 0.01
3,5-NO_2_-Benz-OPh	0.39 ± 0.02	0.053 ± 0.006
3,5-NO_2_-Benz-OHe	0.05 ± 0.008	0.28 ± 0.01

## Data Availability

Data are contained within the article.

## Supplementary Materials

The following supporting information can be downloaded at: https://www.mdpi.com/article/10.3390/ph15091118/s1, Table S1. Characterization of synthesised benzoates.
