# Supplementary material for: Benzoic Acid Derivatives as Prodrugs for the Treatment of Tuberculosis
Source: Pharmaceuticals (Basel). 2022 Sep 7;15(9):1118. doi: 10.3390/ph15091118 (PMC9502840; doi:10.3390/ph15091118)
Supplement: Supplementary file 1 [file pharmaceuticals-15-01118-s001.zip › pharmaceuticals-1840994-supplementary.pdf]

pharmaceuticals

Table S1

Supplementary Data: Ester synthesis and characterization

General ester synthesis procedure. A solution of the appropriate acyl chloride (1.2 eq.) in dichloromethane was added dropwise to a solution of corresponding alcohol and triethylamine (1.2 eq.) in dichloromethane at 0◦C. When the reaction was complete (as assessed by TLC using hexane:ethyl acetate, 5:1 to 1:1,or ethyl acetate as eluent) the reaction mixture was filtered and the filtrate washed successively with 10 mL of distilled water and with 15 mL of saturated sodium bicarbonate solution. The dichloromethane solution was subsequently dried, and the solvent evaporated. The residue was purified by column chromatography (silica gel 60) using hexane: ethyl acetate, 5:1 to 1:1, or ethyl acetate as eluent.

Synthesis of propyl 4-chlorobenzoate. Following the described general procedure, 6 mmol (0,763 mL) of 4-chlorobenzoyl chloride where dissolved in DCM (2,5 mL) and added to a solution in DCM (2,5 mL) of 5 mmol (0,376 mL) of propanol and 6 mmol (0,697 mL) of triethylamine.

Propyl 4-chlorobenzoate - oil; Yield 92%; **^{1}H NMR (300 MHz, $CDCl_3$), δ (ppm):** 7,97 (d, J = 8,8 Hz, 2 H), 7,40 (d, J = 8,8 Hz, 2 H), 4,27 (t, J = 6,8 Hz, 2 H), 1,83-1,74 (m, 2 H), 1,02 (t, J = 7,4 Hz, 3 H) **^{13}C NMR (75 MHz, $CDCl_3$), δ (ppm):** 165, 138, 130, 128, 128, 67, 21, 9, 7. **Infra-red (IR) - (n, cm^{-1})** - 1722,43 (C=O)

Synthesis of hexyl 4-chlorobenzoate. Following the described general procedure, 6 mmol (0,763 mL) of 4-chlorobenzoyl chloride where dissolved in DCM (2,5 mL) and added to a solution in DCM (2,5 mL) of 5 mmol (0,620 mL) of hexanol and 6 mmol (0,697 mL) of triethylamine.

Hexyl 4-chlorobenzoate – brown oil; Yield 76%; **^{1}H NMR (300 MHz, $CDCl_3$), δ (ppm):** 0,92 (3H, t, J=6Hz), 1,37-1,34 (4H, m), 1,41-1,47 (2H, m), 1,74-1,81 (2H, m), 4,33 (2H, t, J=8Hz), 7,43 (2H, d, J=8Hz) 7,98 (2H, d, J=8Hz). **^{13}C NMR (75 MHz, $CDCl_3$), δ (ppm):** 13,99, 22,53, 25,68, 28,64, 31,44, 65,39, 128,65, 128,98, 130,92, 139,21, 165,80. **IR - (n, cm^{-1})** – 1726.29 (C=O)

Synthesis of propyl 3,5-dichlorobenzoate. Following the described general procedure, 6 mmol (1,26 mL) of 3,5-dichlorobenzoyl chloride where dissolved in DCM (2,5 mL) and added to a solution in DCM (2,5 mL) of 5 mmol (0,376 mL) of propanol and 6 mmol (0,697 mL) of triethylamine.

Propyl 3,5-dichlorobenzoate – yellow oil; Yield 92%; **^{1}H NMR (300 MHz, $CDCl_3$), δ (ppm):** 1,05 (3H, t, J=8Hz), 1,76-1,87 (2H, m), 4,31 (2H, t, J=4Hz), 7,55 (1H, d, J=2Hz), 7,92 (2H, s, J=2Hz). **^{13}C NMR (75 MHz, $CDCl_3$), δ (ppm):** 10,44, 22,00, 67,37, 127,96, 132,63, 133,30, 135,22,164,29. **IR - (n, cm^{-1})** - 1732,08 (C=O)

Synthesis of hexyl 3,5-dichlorobenzoate. Following the described general procedure, 6 mmol (1,26 mL) of 3,5-dichlorobenzoyl chloride where dissolved in DCM (2,5 mL) and added to a solution in DCM (2,5 mL) of 5 mmol (0,620 mL) of hexanol and 6 mmol (0,697 mL) of triethylamine.

Hexyl 3,5-dichlorobenzoate – colorless oil; Yield 63%; **^{1}H NMR (300 MHz, $CDCl_3$), δ (ppm):** 0,92 (3H, t, J=8Hz), 1,37-1,36 (4H, m), 1,45-1,41 (2H, m), 1,78 (2H, m, J=8Hz), 4,34 (2H, t, J=8Hz), 7,57 (1H, d, J=4Hz), 7,93 (1H, s, J=2Hz). **^{13}C NMR (75 MHz, $CDCl_3$), δ (ppm):** 13,99, 22,52, 25,62, 28,56, 31,42, 66,01, 127,97, 132,63, 133,32, 138,22, 164,32. **IR - (n, cm^{-1}) –** 1728.22 (C=O)

Synthesis of propyl 3,5-dichlorobenzoate. Following the described general procedure, 6 mmol (1,26 mL) of 3,5-dichlorobenzoyl chloride where dissolved in DCM (2,5 mL) and added to a solution in DCM (2,5 mL) of 5 mmol (0,45 g) of phenol and 6 mmol (0,697 mL) of triethylamine.

Phenyl 3,5-dichlorobenzoate – brown solid; Yield 95%; m.p.:119-121 ºC; **^{1}H NMR (300 MHz, $CDCl_3$), δ (ppm):** 7,23 (2H, d, J=8Hz), 7,31 (1H, d, J=8Hz), 7,47 (2H, t, J=8Hz), 7,68 (1H, s, J=8Hz), 8,05 (2H, d, J=6Hz). **^{13}C NMR (75 MHz, $CDCl_3$), δ (ppm):** 121,42, 126,32, 128,52, 129,63, 131,06, 133,40, 135,53, 150,51, 162,90. **IR - (n, cm^{-1})** - 1735,93 (C=O)

Synthesis of hexyl 2,6-dichlorobenzoate. Following the described general procedure, 6 mmol (1,26 mL) of 2,6-dichlorobenzoyl chloride where dissolved in DCM (2,5 mL) and added to a solution in DCM (2,5 mL) of 5 mmol (0,620 mL) of hexanol and 6 mmol (0,697 mL) of triethylamine.
Hexyl 2,6-dichlorobenzoate – orange oil; Yield 93%; **IR - (n, cm^{-1})** – 1732.08 (C=O)

Synthesis of propyl 4-nitrobenzoate. Following the described general procedure, 3 mmol (0,381 mL) of 4-nitrobenzoyl chloride where dissolved in DCM (2,5 mL) and added to a solution in DCM (2,5 mL) of 2.5 mmol (0,188 mL) of propanol and 3 mmol (0,348 mL) of triethylamine.
Propyl 4-nitrobenzoate – yellow solid; Yield 15%; m.p.:29-31 ºC; **^{1}H NMR (300 MHz, $CDCl_3$), δ (ppm):** 8,29 (dt, J= 9; 2,1Hz, 2H), 8,21 (dt, J= 9; 2,1Hz, 2H), 4,34 (t, J= 6; 9Hz, 2H), 1,76-1,83 (m, 2H), 1,04 (t, J= 6; 9Hz, 3H) **^{13}C NMR (75 MHz, $CDCl_3$), δ (ppm):** 164.84, 150.65, 136.04, 130.80, 123.66, 67.63, 22.16, 10.58

Synthesis of hexyl 4-nitrobenzoate. Following the described general procedure, 3 mmol (0,381 mL) of 4-nitrobenzoyl chloride where dissolved in DCM (2,5 mL) and added to a solution in DCM (2,5 mL) of 2.5 mmol (0,310 mL) of hexanol and 3 mmol (0,348 mL) of triethylamine.
Hexyl 4-nitrobenzoate – yellow oil; Yield 25%; **^{1}H NMR (300 MHz, $CDCl_3$), δ (ppm):** 8,29 (dt, J= 9; 2,1Hz, 2H), 8,21 (dt, J= 9; 2,1Hz, 2H), 4,37 (t, J= 6; 9Hz, 2H), 1,75-1,85 (m, 2H), 1,27-1,51 (m, 2H), 1,04 (t, J= 6; 9Hz, 3H) **^{13}C NMR (75 MHz, $CDCl_3$), δ (ppm):** 164.78, 150.47, 135.89, 130.67, 123.54, 66.26, 31.55, 28.69, 25.78, 22.67, 14.14

Synthesis of propyl 3,5-dinitrobenzoate. Following the described general procedure, 3 mmol (0,631 mL) of 3,5-dinitrobenzoyl chloride where dissolved in DCM (2,5 mL) and added to a solution in DCM (2,5 mL) of 2.5 mmol (0,188 mL) of propanol and 3 mmol (0,348 mL) of triethylamine.
Propyl 3,5-dinitrobenzoate – yellow solid; Yield 60%; m.p.:70-72 ºC; **^{1}H NMR (300 MHz, $CDCl_3$), δ (ppm):** 9,22 (d, J= 3Hz, 1H), 9,16 (d, J= 3Hz, 2H), 4,42 (t, J= 6Hz, 2H), 1,80-1,93 (m, 2H), 1,06 (t, J= 6; 9Hz, 3H) **^{13}C NMR (75 MHz, $CDCl_3$), δ (ppm):** 162.70, 148.84, 134.33, 129.45, 122.44, 68.63, 22.12, 10.52

Synthesis of hexyl 3,5-dinitrobenzoate. Following the described general procedure, 3 mmol (0.631 mL) of 3,5-dinitrobenzoyl chloride where dissolved in DCM (2,5 mL) and added to a solution in DCM (2,5 mL) of 2.5 mmol (0,310 mL) of hexanol and 3 mmol (0,348 mL) of triethylamine.
Hexyl 3,5-dinitrobenzoate – white solid; Yield 44%; m.p.:58-59 ºC; **^{1}H NMR (300 MHz, $CDCl_3$), δ (ppm):** 9,22 (d, J= 3Hz, 1H), 9,16 (d, J= 3Hz, 2H), 4,45 (t, J= 6Hz, 2H), 1,78-1,90 (m, 2H), 1,36-1,49 (m, 2H), 0,95 (t, J= 6Hz, 3H) **^{13}C NMR (75 MHz, $CDCl_3$), δ (ppm):** 162.71, 148.85, 134.35, 134.35, 129.54, 122.42, 67.30, 31.52, 28.66, 25.70, 22.65, 14.10
